# Effects of different blending ratios of lard and chicken fat on growth performance, nutrient utilization, serum lipid metabolism, and tissue fatty acid composition in meat ducks

**DOI:** 10.1016/j.psj.2026.106903

**Published:** 2026-04-07

**Authors:** J.L. Su, G. Tian, K.Y. Zhang, S.P. Bai, X.M. Ding, J.P. Wang, Y. Liu, Y. Xuan, S.S. Li, Q.F. Zeng

**Affiliations:** a Institute of Animal Nutrition, Sichuan Agricultural University, Chengdu, Sichuan, China; b Key Laboratory for Animal Disease-Resistance Nutrition, Ministry of Education, Ministry of Agriculture and Rural Affairs, Sichuan Province 611130, China

**Keywords:** Chicken fat, Lard, Growth performance, Fatty acid composition, Meat ducks

## Abstract

This study aimed to evaluate the effects of lard and chicken fat blending ratios on growth performance, nutrient availability, serum lipid metabolism, and tissue fatty acid composition in meat ducks aged 14 to 42 days. A total of 480 14-day-old meat ducks were randomly divided into 6 treatment groups (8 replicates per group, 10 ducks per replicate), with a total dietary fat addition level of 7.5 %. The dietary ratios of lard to chicken fat were 100:0 (T1), 80:20 (T2), 60:40 (T3), 40:60 (T4), 20:80 (T5), and 0:100 (T6). Results showed that the T3 group (60:40) achieved the lowest feed-to-gain ratio, which significantly outperformed (*P* < 0.05) the pure lard (T1), pure chicken fat (T6), and high-chicken fat groups (T4,T5). Meanwhile, the DM and phosphorus availability in the T3 group were significantly higher (*P* < 0.05) than those in all other groups and the gross energy utilization as well as AME in the T3 group were significantly higher (*P* < 0.05) than those in the T1 and high-chicken fat groups (T4–T6). Serum lipid homeostasis was optimized in the T3 group, maintaining the lowest total cholesterol and LDL-C levels (*P* < 0.05). Fatty acid profiling revealed distinct metabolic partitioning: while leg muscle and subcutaneous fat displayed a sensitive "dietary mirroring" effect, breast muscle maintained lipid homeostasis. These results suggested that the 60:40 lard-to-chicken fat blend constitutes the optimal lipid strategy, which potentially enhances feed efficiency and mineral absorption and optimized systemic lipid clearance and tissue lipid deposition.

China is the world's leading producer and consumer of waterfowl, accounting for over 70 % of global meat duck production (Zeng et al., 2015; da Silva Costa et al., 2023). Under intensive, large-scale rearing systems, modern meat duck breeds, such as the Cherry Valley duck, exhibit rapid growth rates and high metabolic demands, necessitating continuous high-energy supplies (Fan et al., 2008). Benefiting from distinct digestive physiological characteristics—specifically high feed intake, large gastrointestinal capacity, and strong tolerance to fibrous feed—meat ducks are well-suited for the extensive utilization of unconventional, low-energy raw materials to reduce formulation costs (Jamroz et al., 2002; Adeola, 2006; Garaiova et al., 2007). However, the inclusion of such ingredients inevitably dilutes dietary energy density. Consequently, to compensate for this energy deficit and satisfy the demands of rapid growth, increasing the proportion of dietary lipid supplementation has become an essential nutritional strategy (Ravindran et al., 2016; Hu et al., 2019). Nevertheless, recent volatility in global feed ingredient prices, particularly the continuous rise in the cost of traditional vegetable oils like soybean oil, has become a critical bottleneck restricting cost reduction and efficiency improvement in the industry (Ben Hassen and El Bilali, 2022).

Lard and chicken fat are locally abundant and cost-effective animal lipids, representing promising supplemental energy sources for meat duck diets. However, the utility of lard is constrained by its high melting point, attributed to a high content of long-chain saturated fatty acids (SFA), particularly stearic acid (C18:0). Due to the physiological immaturity of young ducks, the secretion of endogenous pancreatic lipase and bile acids is insufficient (Ao and Kim, 2020; Hiżewska et al., 2023). Consequently, high concentrations of SFA readily combine with minerals in the intestinal tract to form insoluble calcium soaps (Tancharoenrat et al., 2013). This saponification process not only results in energy loss but also significantly impairs the bioavailability of essential minerals such as calcium and phosphorus (Atteh, 1984).

To overcome these digestive limitations, leveraging the "synergism" between different lipid sources to enhance SFA utilization has become a key focus in precision nutrition. Chicken fat is rich in linoleic acid (C18:2n6c) and contains a relatively lower proportion of stearic acid (C18:0) compared to lard. These unsaturated fatty acids (UFA) act as co-solvents, reducing the critical micelle concentration and facilitating the incorporation of SFA from lard into mixed micelles for transmembrane transport (Ketels and De Groote, 1989; Wiseman and Salvador, 1991). This synergistic effect not only improves the energy efficiency of lard but also holds potential value for enhancing meat quality. The underlying mechanism suggests that highly absorbed stearic acid (C18:0) can serve as a substrate for endogenous enzymatic desaturation to form oleic acid, thereby optimizing the composition of flavor precursors in poultry meat (Wood et al., 2008).

These findings suggest that blending lard and Chicken fat could further enhance the utilization efficiency and application efficacy of the mixed oil. However, information regarding the optimal blending ratio for meat ducks remains scarce. Therefore, this study was conducted to evaluate the effects of different blending ratios of lard and chicken fat on growth performance, nutrient utilization, serum lipid metabolism, and tissue fatty acid composition in meat ducks.

## Materials and methods

The study was approved by the Animal Care and Use Committee, Sichuan Agricultural University (Ethic Approval Code: SICAUPNZ-202405; Chengdu, China).

### Birds, experimental design, diet, and management

Prior to the formal feeding trial (1 to 14 days of age), all ducklings were fed a basal starter diet (metabolizable energy = 12.11 MJ/kg; crude protein = 20 %). At 14 days of age, a total of 480 male Cherry Valley ducks with similar initial body weights (625 ± 25 g) were selected and randomly assigned to six dietary treatments using a completely randomized design. Each treatment consisted of eight replicates with 10 birds per replicate. A fixed level of 7.5 % supplemental lipid was maintained across all treatments. The specific blending ratios of lard and chicken fat were as follows: T1 (100 % lard), T2 (80 % lard + 20 % chicken fat), T3 (60 % lard + 40 % chicken fat), T4 (40 % lard + 60 % chicken fat), T5 (20 % lard + 80 % chicken fat), and T6 (100 % chicken fat). The lard and chicken fat were sourced from a local market (Chengdu, China), and their fatty acid profiles are presented in Table 1. All experimental diets were formulated to meet or exceed the nutrient requirements for meat ducks recommended by the NRC (1994), as shown in Table 2, and were provided in pelleted form and the fatty acid profiles of these diets are detailed in Table 3. The feeding trial was conducted from 14 to 42 days of age. During the experimental period, birds had ad libitum access to feed and water. Environmental conditions were controlled in accordance with standard commercial rearing practices. Specifically, the room temperature was maintained at 32°C for the first 3 days, gradually decreased to 24°C by day 14, and kept constant thereafter. Relative humidity was maintained at 60-70 %, with a 23L:1D lighting program.

**Table 1:** Fatty acid composition of the lard and chicken fat used in the experiment (% of total Fatty Acids).

Items	Lard	Chicken fat
C12:0	0.07	0.06
C14:0	1.09	0.77
C16:0	24.83	23.00
C16:1	1.35	1.89
C17:0	0.41	0.34
C18:0	12.66	8.14
C18:1n9t	0.15	0.09
C18:1n9c	34.01	33.61
C18:2n6c	22.61	29.24
C18:3n6	0.06	0.10
C18:3n3	1.43	1.63
C20:2	0.39	0.30
C22:0	0.06	0.06
C20:3n6	0.04	0.08
SFA	38.92	32.38
MUFA	35.50	35.59
PUFA	24.82	31.25
U:S ratio^1^	1.53	2.07

1 Unsaturated fatty acid: Saturated fatty acid = UFA:SFA.

**Table 2:** Composition and nutrient contents of the experimental diets (%, air dry basis).

Ingredients	T1(100:0)	T2(80:20)	T3(60:40)	T4(40:60)	T5(20:80)	T6(0:100)
Corn	42.64	42.64	42.64	42.64	42.64	42.64
Soybean meal	25.00	25.00	25.00	25.00	25.00	25.00
Wheat flour	10.00	10.00	10.00	10.00	10.00	10.00
Rice bran meal	6.89	6.89	6.89	6.89	6.89	6.89
Corn gluten meal	4.22	4.22	4.22	4.22	4.22	4.22
Lard	7.5	6.0	4.5	3.0	1.5	0
Chicken fat	0	1.5	3.0	4.5	6.0	7.5
Limestone	1.11	1.11	1.11	1.11	1.11	1.11
Dicalcium phosphate	1.56	1.56	1.56	1.56	1.56	1.56
DL-Methionine	0.11	0.11	0.11	0.11	0.11	0.11
Salt	0.30	0.30	0.30	0.30	0.30	0.30
Choline chloride	0.15	0.15	0.15	0.15	0.15	0.15
Mineral premix^1^	0.50	0.50	0.50	0.50	0.50	0.50
Vitamin premix^2^	0.03	0.03	0.03	0.03	0.03	0.03
Total	100	100	100	100	100	100
Calculated nutrients, %						
Crude protein	18.50	18.50	18.50	18.50	18.50	18.50
Ether extract	7.50	7.50	7.50	7.50	7.50	7.50
Calcium	0.85	0.85	0.85	0.85	0.85	0.85
Available phosphorus	0.40	0.40	0.40	0.40	0.40	0.40
Total lysine	0.88	0.88	0.88	0.88	0.88	0.88
Total methionine	0.40	0.40	0.40	0.40	0.40	0.40
Total threonine	0.68	0.68	0.68	0.68	0.68	0.68
Total tryptophan	0.19	0.19	0.19	0.19	0.19	0.19
Analyzed nutrient, %						
Crude protein^3^	18.27	18.34	18.51	18.49	18.22	18.41
Gross energy^3^, MJ/kg	17.58	17.59	17.62	17.63	17.76	17.72
Ether extract	6.89	6.84	6.97	6.96	6.99	6.87
Calcium	1.06	1.07	0.98	1.00	1.09	1.00
Total phosphorus	0.46	0.48	0.50	0.47	0.50	0.50

1 Mineral premix provides the following per kg of final diet: Fe (FeSO_4_·H_2_O) 80 mg; Cu (CuSO_4_·H2O) 8 mg; Mn (MnSO_4_·H_2_O) 70 mg; Zn (ZnSO_4_·H_2_O) 90 mg; I (KI) 0.4 mg; Se (Na_2_SeO_3_) 0.3 mg. SID-Lys.

2 Vitamin premix provides the following per kg of final diet: vitamin A 8,000 IU; vitamin D 3 2,000 IU; vitamin E 5 mg; vitamin K2 1 mg; vitamin B 1 0.6 mg; vitamin B 2 4.8 mg; vitamin B 6 1.8 mg; vitamin B 12 0.009 mg; niacin 10.5 mg; DL-calcium pantothenate 7.5 mg; folic acid 0.15 mg.

3 With no significant differences in analyzed gross energy and crude protein among all groups (n = 4 replicates per group; one-way ANOVA, *P* > 0.05).

**Table 3:** Compositions of fatty acids of the experimental diets (% of Total Fatty Acids).

Items	T1(100:0)	T2(80:20)	T3(60:40)	T4(40:60)	T5(20:80)	T6(0:100)
C12:0	0.10	0.07	0.07	0.06	0.06	0.06
C14:0	1.09	1.03	0.96	0.90	0.82	0.75
C16:0	24.56	24.18	23.62	23.28	23.23	22.77
C16:1	1.33	1.47	1.57	1.69	1.73	1.83
C17:0	0.41	0.39	0.38	0.37	0.35	0.34
C18:0	12.34	11.52	10.64	9.69	8.79	7.92
C18:1n9t	0.14	0.14	0.13	0.11	0.10	0.09
C18:1n9c	34.00	34.15	33.97	33.91	33.44	33.41
C18:2n6c	23.11	24.21	25.38	27.14	28.57	29.94
C18:3n6	0.06	0.06	0.07	0.08	0.09	0.10
C18:3n3	1.47	1.49	1.52	1.55	1.63	1.64
C20:2	0.42	0.37	0.34	0.33	0.31	0.29
C22:0	0.07	0.06	0.06	0.06	0.06	0.06
C20:3n6	0.04	0.05	0.06	0.07	0.07	0.08
SFA	38.56	37.25	35.72	34.35	33.30	31.90
MUFA	35.48	35.76	35.66	35.71	35.28	35.33
PUFA	25.10	26.18	27.37	29.17	30.66	32.05
U:S ratio^1^	1.57	1.66	1.76	1.89	1.98	2.11

1 Unsaturated fatty acid:Saturated fatty acid = UFA:SFA.

### Data and sample collection

On the morning of d 42, following a 12-h fast (with ad libitum access to water), the fasted body weight and feed consumption of ducks were measured on a per-replicate (cage) basis. Based on these data, average daily gain (ADG), average daily feed intake (ADFI), and feed- to- gain ratio (F/G) were calculated. Mortality was recorded daily throughout the experiment to correct FCR. The European Production Efficiency Factor (EPEF) was calculated using the following formula: EPEF=[Viability ( %) × Final Body Weight(kg)]/[Age(d) × FCR] × 100 %

At d 42, one duck with a body weight close to the average of the cage was randomly selected from each replicate (n = 8). Blood samples (approximately 8 mL) were collected via the jugular vein into non-anticoagulant vacuum tubes. The blood was allowed to clot at room temperature for 3 h and then centrifuged at 3000 × g for 10 min at 4°C. The supernatant serum was separated, aliquoted, and stored at −80°C for subsequent analysis.

### Serum lipid metabolism analysis

The concentrations of total cholesterol (TC), triglycerides (TG), high-density lipoprotein cholesterol (HDL-C), low-density lipoprotein cholesterol (LDL-C), and total bile acids (TBA) in serum were determined using an automatic biochemical analyzer (HITACHI 7180, Tokyo, Japan) with corresponding commercial kits.

### Fatty acid profile analysis

Following blood collection, the ducks were immediately electrically stunned and exsanguinated. Samples of the left subcutaneous fat, breast muscle, and leg muscle were dissected. Visible connective tissues were removed, and the samples were rapidly aliquoted and stored at −80°C for subsequent fatty acid profile analysis.

Fatty acid composition of the diet and tissues (breast muscle, leg muscle, and subcutaneous adipose tissue) was determined by gas chromatography (GC). Lipid Extraction and Derivatization: Approximately 20 g of fresh sample was freeze-dried (FDU-2110; Tokyo Rika Kikai CO., Ltd, Tokyo, Japan) for 60 h and ground into a fine powder. Total lipids were extracted using a chloroform-methanol mixture (2:1, v/v). Subsequently, the extracted lipids were methylated using a boron trifluoride-methanol solution to generate fatty acid methyl esters (FAMEs) following the procedure described by(Sukhija and Palmquist, 1988), while subsequent procedures were conducted according to the protocols described by(Chen et al., 2023).The fatty acid compositions were determined using a gas chromatography analyzer (GS2010 Plus, Shimadzu Co., Ltd, Kyoto, Japan) equipped with a flame ionization detector (FID) and a capillary column (DB-23, 30 m × 0.25 mm × 0.25 μm). The injector and detector temperatures were set at 250°C and 260°C, respectively. Nitrogen was used as the carrier gas. The oven temperature was programmed from an initial 150°C (held for 2 min), increased to 200°C at 5°C/min, and further increased to 230°C at 2°C/min (held for 10 min).

### Nutrient utilization assay

The metabolic trial was conducted from d 42 to d 46. Ninety-six ducks (2 per cage, 8 replicates per treatment) were transferred to metabolic cages and fed the experimental diet supplemented with 0.5 % titanium dioxide (TiO_2_) as an exogenous indicator. Following a 2-day adaptation period, excreta were collected continuously for 72 h. Excreta samples were pooled by cage, cleared of feathers and dander, and stored at −20 °C. The dry matter (DM), nitrogen (N), ether extract (EE), calcium (Ca), and total phosphorus (TP) contents in diets and excreta were analyzed according to(Cunniff, 2023) standard methods. Gross energy (GE) was measured using an oxygen bomb calorimeter (Parr 6400, Parr Instrument Co., Moline, IL, USA). The concentration of TiO_2_ was determined spectrophotometrically according to (Short et al., 1996). Crude protein was calculated as N × 6.25. The apparent nutrient utilization and AME were calculated using the following formulas (Zeng et al., 2022): nutrient utilization (%) = {1 − [(N_e_ × T_d_)/(N_d_ × T_e_)]} × 100, The AME follows: AME = GE_d_ − [(GE_e_) × (T_d_/T_e_)], Where N_e_ and N_d_ represent the nutrient concentration (% DM) in the excreta and diet, respectively; T_e_ and T_d_ represent the TiO_2_ concentration ( % DM) in the excreta and diet, respectively; GE_d_ and GE_e_ represent the gross energy (kcal/kg, DM basis) in the diet and excreta, respectively.

### Statistical analysis

Data were analyzed by one-way analysis of variance (ANOVA) using the General Linear Model (GLM) procedure of SAS 9.4 software (SAS Institute Inc., Cary, NC, USA). The replicate (cage) served as the experimental unit. Differences among treatment means were separated using Tukey’s HSD test. Results are presented as means and standard error of the mean (SEM), with significance defined at *P* < 0.05.

## Results

### Growth performance

As shown in Table 4, no significant differences were observed in final BW or ADG among the treatment groups (*P* > 0.05). The dietary lipid profile significantly affected feed efficiency. The T3 group (60:40) exhibited the lowest F/G ratio, which was significantly lower than that of the pure lard group (T1) and the high-chicken fat groups (T4, T5, and T6) (*P* < 0.05). Similarly, the European Production Efficiency Factor (EPEF) in the T3 group was significantly higher than that in the T1 and T4 groups (*P* < 0.05).

**Table 4:** Effects of blending ratios of lard and chicken fat on growth performance of meat ducks.

Item^1^	T1(100:0)	T2(80:20)	T3(60:40)	T4(40:60)	T5(20:80)	T6(0:100)	SEM	*P*-value
BW^2^ at day 14 (g)	623.69	623.26	624.26	624.29	624.95	624.58	2.04	0.994
BW at day 42 (g)	3427.38	3482.63	3505.38	3380.25	3442.88	3476.38	33.34	0.126
BWG (g)	2803.69	2859.36	2881.11	2755.96	2817.93	2851.80	33.51	0.128
ADG (g)	100.13	102.12	102.90	98.43	100.64	101.85	1.20	0.128
F/G (g/g)	1.907^a^	1.863^b^^c^	1.833^c^	1.876^a^^b^	1.875^a^^b^	1.884^a^^b^	0.01	0.005
ADFI (g)	190.86	190.22	188.57	184.66	188.65	191.79	2.05	0.207
EPI (kg*%/d)	348.30^b^	369.38^a^	374.44^a^	347.24^b^	357.98^a^^b^	360.65^a^^b^	5.96	0.010

1 Values are the means of 8 cages per treatment of 10 ducks per pen (n = 10).

2 BW: body weight; BWG: body weight gain; ADG: Average daily gain; F/G: feed-intake-to-weight-gain ratio. ADFI: average daily feed intake; EPI: European Production Efficiency Factor; SEM, pooled standard error of the mean.

a,b,c Different superscript letters in the same row indicate significant difference (P < 0.05).

### Nutrient utilization

As shown in Table 5, the DM and phosphorus utilization in the T3 group were significantly higher than those in all other treatment groups (*P* < 0.05). Meanwhile, the energy utilization rate and apparent metabolizable energy (AME) in the T3 group were significantly higher than in the T1 and high-chicken fat groups (T4–T6) (*P* < 0.05). Additionally, the digestibility of calcium in the T3 group was significantly higher than in the T1, T4, and T6 groups (*P* < 0.05).

**Table 5:** Effects of blending ratios Lard and Chicken Fat on nutrient utilization of Meat ducks.

Item^1^	T1(100:0)	T2(80:20)	T3(60:40)	T4(40:60)	T5(20:80)	T6(0:100)	SEM	*P*-value
DM, %^2^	71.47^b^	74.14^b^	79.20^a^	72.35^b^	72.55^b^	71.93^b^	1.500	0.007
EE, %	84.71	86.16	90.45	88.78	87.36	85.99	1.692	0.203
Crude protein, %	61.40	66.06	68.00	60.80	63.31	63.45	2.260	0.217
Energy, %	76.34^b^	78.95^a^^b^	82.60^a^	78.26^b^	77.91^b^	77.36^b^	1.354	0.042
AME, kcal/kg	3208^b^	3320^a^^b^	3479^a^	3298^b^	3307^b^	3277^b^	57.273	0.048
TP, %	43.83^b^	44.69^b^	56.90^a^	43.06^b^	48.78^b^	44.35^b^	2.553	0.003
Ca, %	50.45^b^^c^	54.93^a^^b^	59.26^a^	47.52^c^	54.70^a^^b^	51.40^b^^c^	2.228	0.011

1 Values are the means of 8 cages per treatment of 10 ducks per pen (n = 10).

2 AME, apparent metabolizable energy; Ca, calcium; DM, dry matter; EE, ether extract; TP, total phosphorus; SEM, pooled standard error of the mean.

a,b,c Different superscript letters in the same row indicate significant difference (P < 0.05).

### Serum lipid metabolism

As shown in Table 6, Dietary treatments significantly altered the serum lipid profile. No significant differences were observed in triglyceride (TG), HDL-C, or total bile acid (TBA) levels among groups (*P* > 0.05). The T3 group exhibited the lowest serum total cholesterol (TC) concentration, which was significantly lower than that of the T1, T2, T5, and T6 groups (*P* < 0.05). Similarly, the low-density lipoprotein cholesterol (LDL-C) concentration was lowest in the T3 group, significantly lower than in the T4, T5, and T6 groups (*P* < 0.05).

**Table 6:** Effects of Blending ratios Lard and Chicken Fat on serum lipid metabolism of Meat ducks.

Item^1^	T1(100:0)	T2(80:20)	T3(60:40)	T4(40:60)	T5(20:80)	T6(0:100)	SEM	*P*-value
TBA^2^ (umol/L)	19.54	21.98	19.13	20.98	19.69	17.94	3.05	0.953
TG (mmol/L)	0.47	0.47	0.43	0.44	0.45	0.51	0.07	0.960
TC (mmol/L)	3.12^a^	3.22^a^	2.41^b^	2.83^a^^b^	3.47^a^	3.46^a^	0.20	0.005
HDL-C (mmol/L)	1.26	1.30	1.20	1.28	1.43	1.29	0.11	0.790
LDL-C (mmol/L)	0.92^a^^b^	0.91^a^^b^	0.68^b^	0.95^a^	1.04^a^	1.07^a^	0.08	0.031

1 Values are the means of 8 cages per treatment of 10 ducks per pen (n = 10), SEM, pooled standard error of the mean.

2 TBA: total bile acid; TG: triglyceride; TC: total cholesterol; HDL-C: high-density lipoprotein; LDL-C: low-density lipoprotein.

a,b Different superscript letters in the same row indicate significant difference (P < 0.05).

### Breast muscle fatty acid profile

As shown in Table 7, behenic acid (C22:0) levels were significantly higher in the high-lard groups (T1, T2) compared to the high-chicken fat groups (T5, T6) (*P* < 0.05). Stearic acid (C18:0) was highest in the T2 group and lowest in the T4 group (*P* < 0.05). Levels of eicosapentaenoic acid (C20:5n3, EPA) were significantly higher in the T2 group compared to the T4–T6 groups (*P* < 0.05).

**Table 7:** Effects of Blending ratios Lard and Chicken Fat on Breast Muscle Fat fatty acid composition of Meat ducks (% of Total Fatty Acids).

Item^1^	T1(100:0)	T2(80:20)	T3(60:40)	T4(40:60)	T5(20:80)	T6(0:100)	SEM	*p*-Value
C12:0	0.10	0.08	0.02	0.08	0.10	0.02	0.031	0.281
C14:0	0.37	0.37	0.37	0.43	0.35	0.37	0.032	0.575
C16:0	24.05	20.44	24.56	23.82	24.22	24.76	1.256	0.172
C16:1	1.20	1.06	0.79	1.09	0.96	1.04	0.204	0.799
C17:0	0.33	0.36	0.35	0.35	0.34	0.36	0.011	0.330
C18:0	14.74^a^^b^	16.50^a^	15.52^a^^b^	13.78^b^	15.22^a^^b^	14.61^b^	0.584	0.046
C18:1n9t	0.12	0.21	0.12	0.14	0.11	0.09	0.028	0.064
C18:1n9c	23.94	23.40	25.27	24.55	21.63	24.68	1.456	0.566
C18:2n6c	18.81	20.48	18.84	18.68	18.90	19.64	0.520	0.129
C18:3n6	0.06	0.09	0.07	0.08	0.07	0.08	0.011	0.534
C18:3n3	0.43	0.48	0.43	0.53	0.45	0.51	0.036	0.219
C20:2	0.12	0.21	0.12	0.14	0.11	0.09	0.050	0.311
C22:0	0.66^a^	0.71^a^	0.56^a^^b^	0.50^a^^b^	0.40^b^	0.43^b^	0.069	0.013
C20:3n6	1.06	1.21	1.13	1.05	1.05	1.06	0.072	0.596
C23:0	8.05	8.59	8.38	7.48	9.02	8.78	0.540	0.404
C20:5n3	0.40^a^^b^	0.45^a^	0.39^a^^b^	0.35^b^	0.35^b^	0.37^b^	0.026	0.046
C22:6n3	0.64	0.64	0.62	0.53	0.66	0.63	0.056	0.633
SFA	48.30	47.05	49.77	46.44	49.65	49.33	1.502	0.502
MUFA	25.26	24.68	26.18	27.53	22.69	25.81	1.665	0.460
PUFA	22.27	24.31	22.37	22.02	22.39	23.21	0.591	0.083
U:S	0.99	1.09	0.98	1.08	0.92	1.00	0.069	0.509

1 Values are the means of 8 cages per treatment of 10 ducks per pen (n = 10), SEM, pooled standard error of the mean.

a,b Different superscripts within a row indicate a significant difference (*p* < 0.05).

### Leg muscle fatty acid profile

As indicated in Table 8, levels of lauric acid (C12:0) and myristic acid (C14:0) were significantly higher in the pure lard group (T1) compared to the pure chicken fat group (T6) (*P* < 0.05). The accumulation of very-long-chain SFA (C22:0) was significantly suppressed in the T3–T6 groups compared to the T1 and T2 groups (*P* < 0.05). Elaidic acid (C18:1n9t) decreased linearly from T1 to T6 (*P* < 0.05), while linoleic acid (C18:2n6c) increased significantly with rising chicken fat levels (*P* < 0.05). The level of γ-linolenic acid (C18:3n6) in the T2 group was significantly lower than in the T1, T4, T5, and T6 groups (*P* < 0.05). Deposition of α-linolenic acid (C18:3n3) was significantly enhanced in the T5 and T6 groups compared to the T1 group (*P* < 0.01). EPA (C20:5n3) levels were significantly higher in the T1 and T2 groups compared to the T5 group (*P* < 0.05).

**Table 8:** Effects of Blending ratios Lard and Chicken Fat on Leg Muscle Fat fatty acid composition of Meat ducks (% of Total Fatty Acids).

Item^1^	T1(100:0)	T2(80:20)	T3(60:40)	T4(40:60)	T5(20:80)	T6(0:100)	SEM	*p*-Value
C12:0	0.042^a^	0.035^b^	0.038^a^^b^	0.039^a^^b^	0.042^a^	0.035^b^	0.001	0.019
C14:0	0.65^a^	0.59^a^^b^	0.59^a^^b^	0.57^b^^c^	0.59^a^^b^	0.51^c^	0.022	0.003
C16:0	21.17	20.93	20.97	20.56	21.19	20.83	0.209	0.299
C16:1	2.62	2.44	2.49	2.57	2.68	2.61	0.132	0.819
C17:0	0.28	0.30	0.30	0.29	0.29	0.29	0.011	0.905
C18:0	12.32	12.71	12.18	11.69	10.81	11.83	0.513	0.173
C18:1n9t	0.18^a^	0.17^b^	0.16^b^^c^	0.16^c^^d^	0.15^d^	0.13^e^	0.003	<0.001
C18:1n9c	37.12	36.33	37.05	37.89	37.56	35.41	0.795	0.294
C18:2n6c	17.35^c^	18.40^b^	18.17^b^^c^	18.33^b^	19.53^a^	19.82^a^	0.288	<0.001
C18:3n6	0.10^a^	0.08^b^	0.09^a^^b^	0.10^a^	0.10^a^	0.10^a^	0.004	0.014
C18:3n3	0.66^c^	0.66^c^	0.67^c^	0.69^b^^c^	0.76^a^	0.74^a^^b^	0.02	<0.001
C20:2	0.47	0.49	0.48	0.45	0.44	0.50	0.021	0.234
C22:0	0.49^a^	0.5^a^	0.37^b^	0.36^b^	0.27^b^	0.33^b^	0.037	<0.001
C20:3n6	0.51	0.56	0.49	0.51	0.41	0.51	0.042	0.283
C23:0	4.92	4.72	4.87	4.80	4.28	5.28	0.295	0.324
C20:5n3	0.099^a^	0.111^a^	0.089^a^^b^	0.085^a^^b^	0.071^b^	0.087^a^^b^	0.009	0.038
C22:6n3	0.60	0.56	0.60	0.55	0.49	0.64	0.052	0.41
SFA	39.86	39.78	39.31	38.30	37.46	39.10	0.721	0.166
MUFA	39.93	38.95	39.70	40.62	40.38	38.15	0.904	0.400
PUFA	19.78^c^	20.88^b^	20.59^b^^c^	20.70^b^^c^	21.80^a^	22.40^a^	0.317	<0.001
U:S	1.51	1.51	1.54	1.61	1.66	1.55	0.048	0.169

1 Values are the means of 8 cages per treatment of 10 ducks per pen (n = 10), SEM, pooled standard error of the mean.

a-e Different superscripts within a row indicate a significant difference (*p* < 0.05).

### Subcutaneous adipose *fatty acid profile*

As shown in Table 9, levels of lauric acid (C12:0) and myristic acid (C14:0) decreased linearly with increasing chicken fat levels (*P* < 0.05). The content of palmitic acid (C16:0) in the high-lard groups (T1 and T2) was significantly higher than in the T4 and T6 groups (*P* < 0.05). Notably, stearic acid (C18:0) deposition did not follow a linear trend but peaked in the T3 group (6.42 %), which was significantly higher than in all other treatment groups (*P* < 0.05). Linoleic acid (C18:2n6c) showed a significant linear increase with rising dietary chicken fat levels (*P* < 0.05). Regarding n-6 metabolites, the concentrations of γ-linolenic acid (C18:3n6) in the high-chicken fat groups (T4–T6) were significantly higher than those in the high-lard groups (T1–T3) (*P* < 0.05). Similarly, the level of dihomo-γ-linolenic acid (C20:3n6) in the T4 group was significantly higher than in the T1, T2 and T3 groups (*P* < 0.05).

**Table 9:** Effects of Blending ratios Lard and Chicken Fat on Subcutaneous adipose Fat fatty acid composition of Meat ducks (% of Total Fatty Acids).

Item^1^	T1(100:0)	T2(80:20)	T3(60:40)	T4(40:60)	T5(20:80)	T6(0:100)	SEM	*p*-Value
C12:0	0.06^a^	0.05^a^	0.05^b^	0.05^b^	0.05^b^^c^	0.04^c^	0.001	<0.001
C14:0	0.94^a^	0.89^b^	0.84^c^	0.79^d^	0.78^d^	0.72^e^	0.016	<0.001
C16:0	24.12^a^	23.92^a^	23.56^a^^b^	22.85^b^	23.34^a^^b^	22.98^b^	0.265	0.008
C16:1	3.36	3.20	3.08	3.22	3.37	3.28	0.087	0.185
C17:0	0.22	0.22	0.22	0.22	0.21	0.21	0.006	0.353
C18:0	5.73^b^^c^	5.94^b^	6.42^a^	5.53^c^	5.58^b^^c^	5.68^b^^c^	0.128	<0.001
C18:1n9t	0.24	0.24	0.23	0.24	0.21	0.206	0.013	0.252
C18:1n9c	47.14	46.57	46.32	46.59	45.44	45.12	0.519	0.074
C18:2n6c	15.93^d^	16.52^c^^d^	17.03^c^	17.89^b^	18.72^a^	19.45^a^	0.275	<0.001
C18:3n6	0.09^b^	0.09^b^	0.10^b^	0.12^a^	0.11^a^	0.12^a^	0.005	<0.001
C18:3n3	0.92	0.93	0.94	0.94	0.94	0.98	0.014	0.076
C20:2	0.27	0.29	0.27	0.27	0.27	0.27	0.008	0.496
C22:0	0.15	0.14	0.15	0.17	0.14	0.14	0.010	0.322
C20:3n6	0.13^c^	0.14^b^^c^	0.15^b^^c^	0.19^a^	0.17^a^^b^	0.17^a^^b^	0.011	0.004
C20:5n3	0.01	0.01	0.01	0.01	0.01	0.02	0.004	0.278
C22:6n3	0.01	0.01	0.01	0.01	0.01	0.01	0.001	0.551
C23:0	0.19	0.20	0.21	0.24	0.21	0.21	0.017	0.408
SFA	31.40^a^	31.43^a^	31.21^a^	30.05^b^	30.35^a^^b^	29.98^b^	0.386	0.017
MUFA	50.74	50.00	49.62	50.05	49.02	48.60	0.566	0.126
PUFA	17.36^d^	17.99^c^^d^	18.51^c^	19.43^b^	20.22^a^^b^	21.01^a^	0.297	<0.001
U:S	2.17^b^	2.17^b^	2.18^b^	2.32^a^	2.28^a^^b^	2.33^a^	0.042	0.011

1 Values are the means of 8 cages per treatment of 10 ducks per pen (n = 10), SEM, pooled standard error of the mean.

a-e Different superscripts within a row indicate a significant difference (*p* < 0.05).

### Correlation analysis of fatty acid deposition

**Fig. 1:**
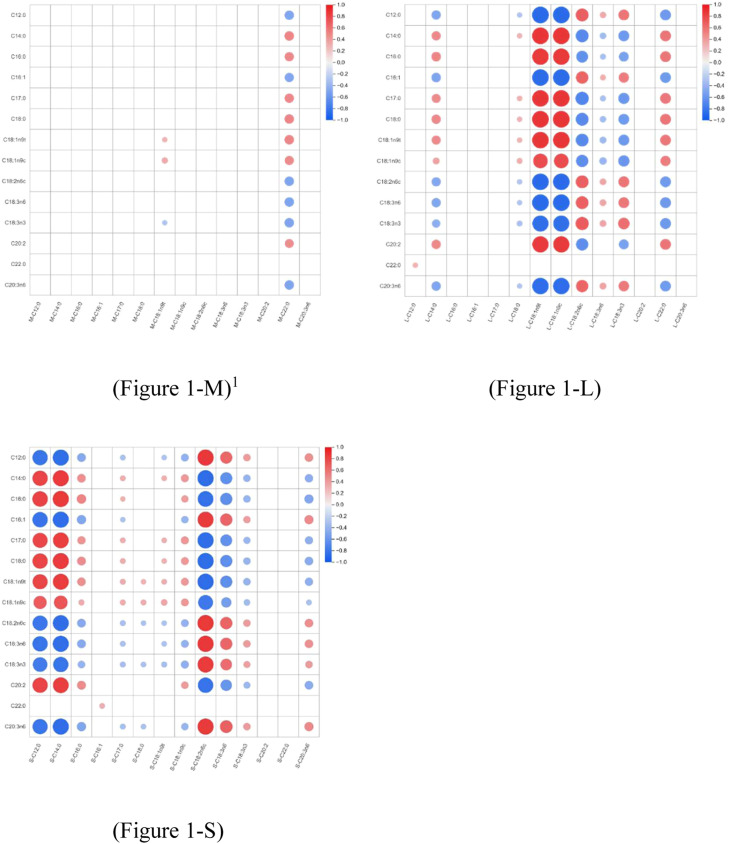
Spearman correlation heatmap between dietary fatty acid profiles and the fatty acid compositions of various tissues. ^1^The vertical axis represents the dietary fatty acid species; the horizontal axis represents the fatty acid compositions within the sampled tissues. L, leg muscle; M, breast muscle; S, subcutaneous fat.

Spearman correlation analysis revealed distinct tissue-specific patterns (Fig. 1). The breast muscle (Fig. 1-M) exhibited a largely homeostatic profile with weak correlations for most fatty acids. In contrast, leg muscle (Fig. 1-L) and subcutaneous fat (Fig. 1-S) displayed extensive positive correlations between dietary intake and tissue deposition for C12:0, C14:0, and C18:2n6c. Furthermore, dietary C18:2n6c was positively correlated with its metabolite C18:3n6 in both tissues, while exhibiting a negative correlation with C22:0 in leg muscle and C18:0 in subcutaneous fat. Notably, dietary C18:1n9c showed a positive correlation with C18:0 in both leg muscle and subcutaneous fat.

## Discussion

In the present study, the specific combination of 60 % lard and 40 % chicken oil (T3) yielded the optimal physiological outcome, driven by a precise "synergistic equilibrium" (U:S ratio ≈ 1.76). This formulation not only maximized growth performance and nutrient utilization (dry matter, energy, and phosphorus) through enhanced micellar solubilization but also optimized serum lipid homeostasis by maintaining the lowest cholesterol levels. Crucially, a distinct tissue-specific metabolic partitioning was revealed: while subcutaneous fat and leg muscle exhibited a sensitive "dietary mirroring" effect indicative of direct deposition, breast muscle maintained a strict homeostatic stability regardless of the dietary lipid source.

The composition of the dietary fatty acid profile is a core factor regulating energy utilization efficiency in waterfowl (Saleh et al., 2021). In the present study, the specific 60:40 blend of lard and chicken oil (T3) yielded superior production performance, mechanistically supported by maximized nutrient utilization. Specifically, the T3 group achieved peak levels of AME and availability for dry matter and phosphorus. According to the synergistic absorption theory, the introduction of unsaturated fatty acids (UFA) from chicken oil reduces the critical micelle concentration and effectively inhibits intestinal saponification, thereby facilitating the entry of stearic acid into mixed micelles and enhancing its diffusion efficiency across the intestinal unstirred water layer (Tancharoenrat et al., 2013; Hu et al., 2019). This physiological "synergistic equilibrium" (U:S ≈ 1.76) fundamentally underpins the observed growth advantage. However, as highlighted by(Ravindran et al., 2016), the fatty acid composition of lipid sources can vary widely. When comparing our lipid sources to the typical profiles tabulated in their review, the major saturated fatty acids—such as palmitic acid (C16:0) and stearic acid (C18:0) in both lard and chicken fat—aligned well. However, certain other components, such as linoleic acid (C18:2n6c) in both sources, showed notable deviations from conventional profiles. It should be noted as a limitation that the optimal 60:40 ratio identified in this study is inherently tied to the specific fatty acid profiles of these exact lipid batches. Natural variations in fat sources, such as using different batches of lard and chicken fat or alternative lipid sources, could alter this optimal target ratio in practical applications.

Beyond nutrient absorption, the observed growth advantage also stems from more efficient systemic lipid homeostasis regulation. Notably, despite having the highest AME, the T3 group exhibited significantly the lowest serum total cholesterol (TC) and low-density lipoprotein cholesterol (LDL-C) levels. This characteristic of "high energy intake, low lipid retention" suggests that lipid balance was effectively shifted from blood circulation to peripheral tissue clearance. Potential mechanisms may involve the activation of metabolic pathways and the inhibition of endogenous synthesis. On one hand, optimized lipid digestion and absorption efficiency significantly upregulated the expression of key metabolic genes. Studies have shown that improved lipid absorption can activate *PPARα* and *lipoprotein lipase (LPL)* in the liver and adipose tissue, accelerating the transport and clearance of triglycerides carried by very-low-density lipoprotein (VLDL) from the blood to tissues (Ge et al., 2019; Ao and Kim, 2020; Wang et al., 2024). In summary, the physiological state of efficient absorption and rapid turnover in the T3 group effectively alleviated lipid metabolic stress in the circulatory system.

Compared to other tissues, the breast muscle did not exhibit similar dietary fingerprint signatures for major fatty acids (C18:2n6c, C18:3n3). The correlation analysis further confirmed that even for medium-chain fatty acids (C12:0, C14:0) which fluctuated drastically in the diet, their correlation coefficients with corresponding components in the breast muscle were far lower than those in leg muscle and subcutaneous fat, indicating that the breast muscle possesses strong metabolic buffering capacity. This difference is attributed to fundamental differences in the lipid forms present in different muscle types (Zhang et al., 2022). As a low-fat glycolytic muscle, the lipids in breast muscle primarily consist of membrane phospholipids. The fatty acid composition of membrane phospholipids is subject to strict homeostatic regulation to maintain specific membrane fluidity and cellular function, thereby demonstrating stronger structural conservation(Fan et al., 2020; Guo et al., 2020).

Conversely, the leg muscle demonstrated a high dependency on dietary lipid sources. As the proportion of chicken oil increased, lauric acid (C12:0) and myristic acid (C14:0) decreased linearly, while linoleic acid (C18:2n6c) and α-linolenic acid (C18:3n3) increased linearly, highly reflecting the dietary fatty acid profile. This was strongly verified in the correlation analysis: dietary C12:0 and C14:0 showed extremely significant positive correlations with their corresponding components in the leg muscle, displaying a characteristic of direct deposition with almost no modification. Notably, the massive influx of linoleic acid(C18:2n6c) triggered a significant "Mass Action Effect" in the leg muscle: the significant elevation of γ-linolenic acid (C18:3n6) suggests a potential upregulation of endogenous Δ6-desaturase activity driven by excess substrate. This was also captured by the correlation data, where dietary C18:2n6c presented a significant positive correlation with leg muscle C18:3n6, confirming the direct driving role of substrate concentration on downstream metabolites (Takić et al., 2024). Furthermore, trans-oleic acid (C18:1n9t), acting as a specific fingerprint for lard, showed a strict linear decrease; serving as an exogenous biomarker unaffected by metabolic masking that effectively validates the precision of the dietary lipid gradient and the universality of tissue absorption. This point was also validated by physiological data in a recent study by (Li et al., 2024) regarding muscle fatty acid profiles and meat quality characteristics in different waterfowl strains.

However, strong metabolic regulation targeting long-chain fatty acids was observed in both muscle tissues. In the high-lard groups (T1 and T2), excessive C18:0 intake combined with a deficiency in dietary UFA likely shifted the substrate flux, potentially exceeding the instantaneous desaturation capacity of the SCD1 enzyme. The resulting "metabolic congestion" forced unconverted stearic acid to spill over into the ELOVL-mediated elongation pathway, synthesizing very-long-chain saturated fatty acids (Liu et al., 2019; Guo et al., 2020). Leg muscle data provided solid evidence for this mechanism: C22:0 content remained high in the T1 and T2 groups, whereas it dropped precipitously to 0.370 % in the T3 group. The correlation analysis provided evidence for this "unclogging" mechanism: dietary C18:2n6c were significantly negatively correlated with C22:0 in the leg muscle. This statistically confirms that as dietary unsaturation increased, the aberrant elongation of long-chain saturated fatty acids was effectively inhibited. The significant reduction of C22:0 in both muscle tissues indicates that the optimized U:S ratio of the T3 group "unclogged" the systemic metabolic bottleneck, effectively diverting C18:0 to the desaturation pathway to generate oleic acid, rather than generating the more toxic C22:0 (Wood et al., 2008; Li et al., 2024).

Similarly, as the proportion of chicken oil increased, eicosapentaenoic acid (EPA, C20:5n3) in both breast and leg muscles showed a significant downward trend. This confirms that high levels of linoleic acid in the high-chicken oil groups exerted competitive pressure on the shared Δ6-desaturase, preempting the opportunity for n-3 precursors to be converted into EPA (Fan et al., 2020; Kowalska et al., 2020). Therefore, although the breast muscle maintained homeostasis for major dietary fatty acids, its specific metabolites (C22:0 and EPA) remained sensitive to systemic regulation driven by enzyme-substrate competition, similar to the leg muscle.

Finally, the fatty acid composition of subcutaneous adipose tissue serves as the most sensitive biological indicator of systemic net lipid absorption(Kowalska et al., 2020). In this study, as the proportion of chicken oil increased, lauric acid, myristic acid, and palmitic acid showed significant linear decreases, while linoleic acid increased linearly. This typical "non-selective deposition" characteristic is highly consistent with findings in broilers (Saleh et al., 2021) and Peking ducks (Chen et al., 2023). Furthermore, the significant increase in γ-linolenic acid in the high-chicken oil groups further confirms that linoleic acid acted as a substrate to drive the "Mass Action Effect" of endogenous desaturases (Kartikasari et al., 2012; Fan et al., 2020; Takić et al., 2024), an effect particularly pronounced in storage tissues.

Critically, despite the lower dietary stearic acid content in the T3 group (10.64 %) compared to the T1 group (12.34 %), C18:0 deposition in subcutaneous fat reached a significant peak (6.424 %). This biological paradox of "low intake, high deposition," corroborated by the positive correlation between dietary C18:1n9c and tissue C18:0,provides metabolic endpoint evidence that the T3 formulation maximized the absorption efficiency of long-chain saturated fatty acids through synergistic action. It is worth noting that although dietary linoleic acid and tissue stearic acid exhibited a statistical negative correlation in the Spearman heatmap, this relationship is largely confounded by the stoichiometric reduction in substrate (C18:0) intake resulting from the substitution of lard (i.e., the "dilution effect"). However, the dilution effect alone cannot explain the observed paradox. Specifically, while the T1 group possessed slightly higher oleic acid levels, its deficiency in linoleic acid failed to cope with the highest stearic acid load, limiting absorption. In contrast, concurrent with the gradient reduction in stearic acid substrate concentration in the T3 group, the introduction of high levels of linoleic acid effectively established an optimal "oleic-linoleic-stearic" ternary synergistic system. This implies that oleic acid alone is insufficient to maximize micellization efficiency; rather, linoleic acid exerts an indispensable "solubilization effect" by enhancing the fluidity and hydrophobic core volume of mixed micelles, thereby facilitating stearic acid transport across the intestinal unstirred water layer. (Rodriguez-Sanchez et al., 2021) demonstrated that unsaturated diets significantly enhanced the ileal digestibility of saturated fatty acids by assisting mixed micelle formation, thereby supporting our observation that the enrichment of linoleic acid facilitates the absorption and subsequent deposition of stearic acid.

## Conclusions

In conclusion, this study identifies the 60:40 lard-to-chicken oil blend (T3) as the optimal lipid strategy, which significantly improves feed efficiency and dietary nutrient utilization as well as AME, mechanistically driven by a "synergistic equilibrium" (U:S ≈ 1.76). Moreover, the distinct tissue partitioning characterized by homeostatic breast muscle versus responsive leg and subcutaneous adipose tissues provides a theoretical basis for precision feed formulation. This strategic blending of lipid sources valorizes animal fat by-products and offers insights into ideal fatty acid patterns in poultry production.

## CRediT authorship contribution statement

**J.L. Su:** Writing – original draft, Visualization, Validation, Software, Project administration, Methodology, Investigation, Formal analysis, Data curation. **G. Tian:** Supervision, Resources, Conceptualization. **K.Y. Zhang:** Writing – review & editing, Supervision, Methodology, Conceptualization. **S.P. Bai:** Writing – review & editing, Supervision, Project administration, Funding acquisition, Conceptualization. **X.M. Ding:** Writing – review & editing, Supervision, Resources, Investigation, Conceptualization. **J.P. Wang:** Writing – review & editing, Supervision, Resources, Project administration, Investigation, Conceptualization. **Y. Liu:** Supervision, Resources, Project administration, Methodology, Conceptualization. **Y. Xuan:** Software, Methodology, Investigation. **S.S. Li:** Supervision, Resources, Methodology, Investigation. **Q.F. Zeng:** Writing – review & editing, Supervision, Resources, Funding acquisition, Conceptualization.

## Disclosures

No conflicts of interest exist in the submission of this manuscript, and the manuscript has been approved by all authors for publication.
